# Survival and mortality of preterm neonates in a neonatal intensive care unit in Northern Ethiopia: a retrospective cohort study

**DOI:** 10.1038/s41598-021-04521-z

**Published:** 2022-01-12

**Authors:** Gdiom Gebreheat, Hirut Teame

**Affiliations:** 1 Department of Nursing, College of Medicine and Health Sciences, Adigrat University, Adigrat, Ethiopia; 2 Department of Public Health, College of Medicine and Health Sciences, Adigrat University, Adigrat, Ethiopia

**Keywords:** Health care, Medical research

## Abstract

The purpose of this study was to assess the predictors of preterm neonatal survival in a neonatal intensive care unit (NICU). A cohort study was conducted retrospectively on 1017 preterm neonates using medical records from January 2014 through December 2018. The Kaplan–Meier model was used to estimate mean survival time and cumulative survival probability. Furthermore, Multivariable Cox regression analysis was run to identify predictors of preterm neonatal mortality using an adjusted hazard ratio (AHR) at P < 0.05 and 95% confidence interval (CI). During the follow-up period in the NICU, the mean survival time of the preterm neonates was 47 (95% CI (43.19–48.95)) days. Compound presentation (AHR = 2.29, 95% CI (1.23–4.24)), perinatal asphyxia (AHR = 2.83, 95% CI (1.75–4.58)), respiratory distress syndrome (AHR = 3.01, 95% CI (1.80–5.01)), 1-min APGAR score (AHR = 0.78, 95% CI (0.62–0.98)), and birth weight (AHR = 0.32, 95% CI (0.17–0.58)) were found to be significant predictors of time to preterm neonatal mortality. In conclusion, the survival probability of preterm neonates showed a considerable decrement in the first week of life. Fetal presentation, gestational age, birth weight, 1-min APGAR score, perinatal asphyxia and respiratory distress syndrome found as independent predictors of preterm neonatal mortality.

## Introduction

According to World Health Organization (WHO), preterm birth is defined as babies born alive before 37 weeks of pregnancy are completed. Each year, 15 million babies are born preterm in the world. Mortality is higher among preterm neonates than those of term^1–7^. Globally, prematurity is the leading cause of under-five children mortality^8^. Hence, preterm neonatal mortality has continued as an important public health issue.

In a 2014 systematic review of global preterm births, the estimated regional preterm birth rates were ranged from 13.4 in North Africa to 8.7% in Europe. Asian and sub-Saharan African countries were contributed to 81.1% of preterm births worldwide. The review also showed that the magnitude of preterm mortality is increasing as years pass by regardless of geographic and economic disparity among nations^9^. The improvements in pregnancy and delivery care have resulted in increased survival of the fetus to delivery and attempted resuscitation at lower gestational ages, but with a higher risk of mortality among these more preterm neonates. For instance, in Hubei Province, China, the incidence of preterm birth had increased from 56.7 to 105.2 per 1000 live births from 2001 to 2012^10^.

The common predictors of preterm neonatal deaths are gestational age, birth weight, lower APGAR score, respiratory distress syndrome, sepsis, pneumonia, meningitis and asphyxia^1,11–15^. As of 2014, Ethiopia was among the top ten countries with the highest number of preterm births globally. In the nation, 320,000 babies are born prematurely every year. Today, 1 out of 10 babies are dying because of prematurity^16^. Likewise, studies conducted in Ethiopia estimate the magnitude of preterm mortality lies between 8.1 and 28.8%^13,14,17,18^. Preterm deaths have risen over recent years, and the reduction of preterm mortality is a priority of the Ethiopian government^19^. Even though neonatal mortality has decreased from 49 deaths per 1000 live births in 2005 to 30 deaths per 1000 births in 2019, still 42% of under-five deaths are occurring in the neonatal period^9,19^. Similarly, the Ethiopia Demographic and Health Survey (EDHS) (2016) report revealed that 1 in every 35 under-five children dies within the neonatal period^20^. For this reason, the Ethiopian Ministry of Health developed the first comprehensive National Child Survival Strategy (2005–2015) in 2005 which was being implemented as part of the 3rd and 4th Health Sector Development Program (HSDP) cycles. Currently, the country has revised child survival and the long-term strategy (2015–2020) with a vision to end all preventable child deaths by 2030^21^. As part of the strategy, studies have been conducted on survival status and predictors of preterm neonatal death in different regions of the country. For example, a study conducted at the University of Gondar Comprehensive Specialized Hospital indicated that 96.71% of preterm neonates survived at the end of the first day of admission to the neonatal intensive care unit (NICU). According to the study, home delivery, hyaline membrane disease, gestational age, cry immediately at birth, kangaroo mother care, presence of jaundice and hypoglycemia at admission were found to be significant predictors of time to death for preterm neonates^13^.

Despite the evidence that identified predictors of preterm neonatal survival in Jimma and Gondar, little is known in the other regions of Ethiopia.

Therefore, addressing the limitation of data regarding the predictors of preterm neonatal survival in ACSH, the biggest teaching and referral hospital in the Tigray region of Ethiopia, will help policymakers and other concerned bodies in planning and resources allocation to the region. The purpose of this study was to assess the predictors of preterm neonatal survival in NICU of ACSH in northern Ethiopia.

## Methods

### Study setting, design and population

This retrospective cohort study was conducted in the biggest teaching and referral hospital of the Tigray region of Ethiopia, ACSH. This hospital provides in-patient and out-patient services across various departments and wards. NICU is one of the units under the pediatric ward which offers intensive care and invasive procedures for neonates in an inpatient base. All neonates with a gestational age of less than 37 weeks and admitted to the NICU at ACSH between January 1, 2014, and December 31, 2018, were included in this retrospective cohort study. However, neonates with incomplete medical records were excluded. We declared incomplete medical records if more than 5% of the variables in the checklist are missed in the medical records. During the study period, a total of 1253 preterm neonates were admitted to the NICU in ACSH, of which we excluded 236 due to incomplete medical records.

### Definitions of variables

The variables of interest included for preterm neonatal mortality were characteristics of the neonates at delivery (mode of delivery, fetal presentation, sex, APGAR score at 1st and 5th minute, gestational age and birth weight) and medical morbidities (hypothermia, neonatal sepsis, perinatal asphyxia, hypoglycemia, meningitis, jaundice, respiratory distress syndrome, HIV exposure, seizure, birth trauma and others). The diagnosis and management of neonatal problems are performed according to the National Neonatal Intensive Care Unit Management Protocol, which is usually adopted from WHO recommendations^22^. In this study, therefore, any medical diagnosis of neonates adopted as exactly written/stated in the neonate’s medical record chart by the physician or others who were in charge of diagnosis.

### Data collection procedure and quality assurance

A structured and pretested checklist was prepared in English after reviewing related research articles, Demographic and Health Survey (DHS) documents and recommendations^1,2,7,12,13,15,23–32^. It primarily focused on preterm neonatal baseline data and morbidities. The checklist was pretested on 100 medical records in the NICU of Adigrat General Hospital, with subsequent revisions to the checklist. Data were extracted from preterm neonatal charts (including documented admission and discharge diagnosis, and laboratory data) and Health Management Information Systems (HMIS) booklet by six trained Baccalaureate Degree (BSc) nurses, and supervised by two Master of Science (MSc) holders in pediatric nursing.

### Data analysis

Data were entered into Epi-data version 3.5 and exported to STATA version 14. In univariate analysis, Kaplan–Meier curves were used to estimate mean survival time and cumulative probability of survival. A Log-rank test was also used to compare the statistical survival difference between categories of different explanatory variables. Next, proportional hazard assumption was checked both graphically and hypothesis test called Schoenfeld residuals test. The Schoenfeld residuals test (global test) for all variables and log–log plot for categorical variables showed that proportional hazard assumptions were met. In bivariate analysis, variables significant at P < 0.05 level were included for adjustment in the final Cox regression analysis to identify the independent predictors of mortality. Then, a better model was selected using the Log-likelihood Ratio (LR) test and Akaike Information Criterion (AIC). Accordingly, we selected the model that included fetal presentation, 1- and 5-min APGAR score, gestational age, birth weight, perinatal asphyxia, and respiratory distress syndrome as predictors of preterm neonatal mortality. Also, multi-collinearity was checked using the variance inflation factor for predictor variables. The goodness of model fitness was also checked by Nelson Aalen cumulative hazard function against Cox-Snell residual. In multivariate Cox regression analysis, the association between variables was summarized by using AHR, and P < 0.05 level of significances at 95% CI. In the analysis model, continuous variables such as gestational age (weeks), APGAR score (0–10) and birth weight (kg) were analyzed as numeric variables, whereas respiratory distress syndrome and perinatal asphyxia were managed as categorical variables.

### Ethical statement

All methods were performed in accordance with the relevant guidelines and regulations. Ethical approval and clearance were secured by the Health Research Ethics Review Committee (HRERC), College of Health Sciences, Mekelle University with reference code ERC1375/2019 to conduct the study in NICU at ACSH. The HRERC has also waived the requirement for informed consent to access the data from the medical records. Moreover, medical records were not exposed to any other third party.

## Results

### Characteristics of neonates at delivery

Based on the available medical records, a total of 1253 preterm neonates were admitted to the NICU in ACSH. A total of 1017 (81.1%) preterm neonates were included in the final study population after excluding 236 (18.8%) ineligible medical records. The median gestational age of the preterm neonates was 34 (IQR 33–35.28) weeks. The most common type of fetal presentation was vertex which accounted for 792 (77.8%) of all deliveries while nearly half (52.2%) of the neonates were born via cesarean section (CS). In this study, 804 (79.0%) of the preterm neonates had records of the first minute APGAR score, and only 15 (1.8%) of them were scored 0–3. The mean birth weight of the neonates was 1.8 ± 0.4 kg. Of all the study participants, only 27 (2.6%) of the neonate had birth trauma (Table 1).

**Table 1:** Clinical characteristics of preterm neonates admitted in NICU at ACSH, Northern Ethiopia.

Characteristics	Died, n (%)	Alive, n (%)
**Gestational age (weeks)**		
< 32	88 (47.3)	98 (52.7)
32–34.6/7	33 (8.3)	363 (91.7)
35–36.6/7	28 (6.4)	407 (93.6)
**Fetal presentation**		
Vertex	119 (15)	673 (85)
Breech	15 (1)	135 (99)
Footling	0 (0.0)	18 (100)
Compound	15 (26.3)	42 (73.7)
**Mode of delivery**		
Cesarean section	64 (12)	467 (88)
Vaginal delivery	85 (17.5)	401 (82.5)
**Sex**		
Male	76 (15)	431 (85)
Female	73 (14.3)	437 (85.7)
**APGAR score at minute 1 (n = 804)**		
0–3	6 (40)	9 (60)
4–6	74 (22.2)	259 (77.8)
7–10	39 (8.5)	417 (91.5)
**APGAR score at minute 5 (n = 801)**		
0–3	3 (33.3)	6 (66.7)
4–6	55 (50.9)	53 (49.1)
7–10	61 (8.9)	623 (91.1)
**Birth weight (kg)**		
< 1	24 (66.7)	12 (33.3)
1 to < 1.5	67 (32.3)	140 (67.7)
1.5 to < 2.5	57 (8.3)	627 (91.7)
≥ 2.5	1 (1.1)	89 (98.9)
**Birth trauma**		
Yes	3 (11.1)	24 (88.9)
No	146 (14.7)	844 (85.3)

### Medical morbidities and death in preterm neonates

Overall, only 78 (7.6%) of the preterm neonates had no record of any medical morbidity. Furthermore, hypothermia, neonatal sepsis and respiratory distress syndrome were recorded as frequent morbidities among admitted preterm neonates, 642 (63.1%), 600 (59%) and 390 (38.3%), respectively (Table 2).

**Table 2:** Commonly reported medical morbidities and interventions indicated to preterm neonates.

Characteristics	Died, N (%)	Alive, N (%)
**Medical morbidities**		
Hypothermia		
Yes	88 (13.7)	554 (86.3)
No	61(16.3)	314 (83.7)
Neonatal sepsis		
Yes	106 (17.7)	494 (82.3)
No	43 (10.3)	374 (89.7)
Respiratory distress syndrome		
Yes	120 (30.8)	270 (69.2)
No	29 (4.6)	598 (95.4)
Hypoglycemia		
Yes	15 (14.3)	90 (85.7)
No	134 (14.7)	778 (85.3)
Jaundice		
Yes	19 (18.1)	86 (81.9)
No	130 (14.2)	782 (85.8)
Perinatal asphyxia		
Yes	31 (39.7)	47 (60.3)
No	118 (12.6)	821 (87.4)
HIV exposed		
Yes	3 (25.0)	9 (75.0)
No	144 (11.7)	861 (88.3)
Seizure		
Yes	6 (50.0)	6 (50.0)
No	143 (14.3)	862 (85.7)
Meningitis		
Yes	6 (66.7)	3 (33.3)
No	141 (14.0)	867 (86.0)
Others		
Yes	22 (33.3)	44 (67.7)
No	125 (13.1)	826 (86.9)
**Type of intervention**		
Cardiopulmonary resuscitation	70 (55.6)	56 (44.4)
Corticosteroid	18 (18.2)	81 (81.8)
Antibiotic	127 (17.1)	614 (82.9)

The proportion of death among preterm neonates was 14.6% (95% CI (12.6–16.9%)). The associated morbidities with preterm neonatal death were respiratory distress syndrome (29%), neonatal sepsis (25%), hypothermia (21%), perinatal asphyxia (7%), jaundice (4%) and hypoglycemia (4%).

### Survival status of preterm neonates

A total of 1017 preterm neonates were followed for a minimum of 1 day and a maximum of 60 days with a median follow-up period of 6 days. The date of birth was referred to as a time of origin. The event of our study was the occurrence of neonatal death during the period of hospitalization. Survival was stated as neonates who remained alive during their period of hospitalization, discharged against medical advice, discharged with improvement, or referred to other health institutions. In sum, 868 (85.3%) of the neonate survived while 149 (14.6%) died. Furthermore, among the neonates admitted to NICU, 120 (11.7%) died in the first 5 days of their life.

**Figure 1:**
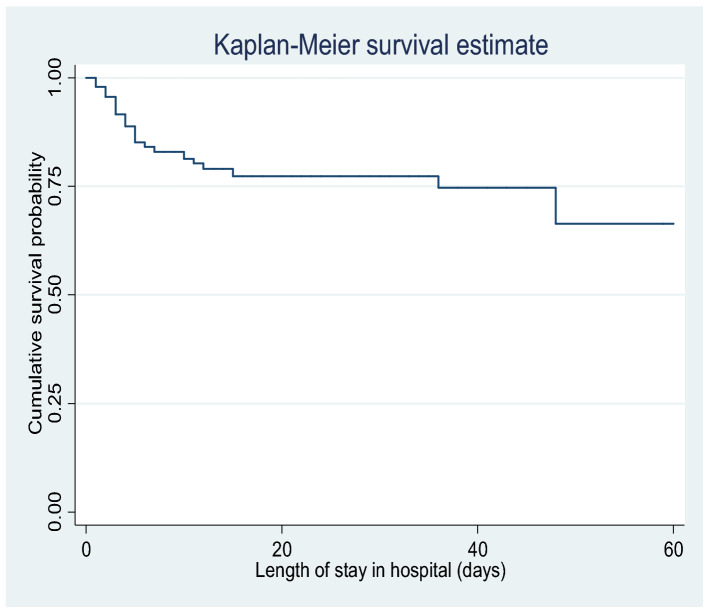
Kaplan–Meier estimates of cumulative survival probability of preterm neonates in NICU at ACSH, Northern Ethiopia, from 2014 to 2018.

The cumulative probability of survival at the 1st, 7th, and 15th day of life was 97.9%, 82.9% and 77.2%, respectively with a considerable difference across various variables (Fig. 1). Time to death was stated as the time from date of birth to the occurrence of death during their period of hospitalization. The survival time was measured in days. Overall, during the follow-up period in the NICU, the mean survival time of the preterm neonates was 47.0 (95% CI (43.1–48.9)) days.

### Predictors of mortality in preterm neonates

In bivariate analysis, gestational age, birth weight, fetal presentation, first and fifth minute APGAR score, respiratory distress syndrome and perinatal asphyxia showed significant statistical association with preterm neonatal death. After adjustment for confounding variables, the fifth minute APGAR score was not continued as a predictor variable of death (Table 3).

**Table 3:** Bivariate and multivariate Cox-proportional hazard regression on predictors of mortality for preterm neonates.

Variables	CHR (95% CI)	AHR (95% CI)	P-value
**Fetal presentation**			
Vertex	1	1	
Breech	0.66 (0.38–1.13)	0.9 (0.51–1.61)	0.74
Footling	5.75e−16 (0–0.0)	1.91e−16 (0–0.0)	1
Compound	2.2 (1.28–3.77)	2.29 (1.23–4.24)	0.009*
Gestational age (weeks)	0.72 (0.68–0.76)	0.87 (0.79–0.95)	0.003*
1st minute APGAR score	0.62 (0.54–0.70)	0.78 (0.62–0.98)	0.04*
5th minute APGAR score	0.60 (0.54–0.67)	0.95 (0.79–1.15)	0.64
Birth weight (kg)	0.09 (0.06–0.14)	0.32 (0.17–0.58)	< 0.001*
**Respiratory distress syndrome**			
Yes	5.69 (3.77–8.59)	3.01 (1.80–5.01)	< 0.001*
No	1	1	
**Perinatal asphyxia**			
Yes	3.06 (2.05–4.55)	2.83 (1.75–4.58)	< 0.001*
No	1	1	

*CHR* crude hazard ratio, *AHR* adjusted hazard ratio.

*Statistical significance of variables at P < 0.05.

The risk of preterm neonatal mortality was approximately 2.3 times higher among those whose presentation was compound in comparison with those whose presentation was vertex, (AHR = 2.29, 95% CI (1.23–4.24)). Neonates who were also diagnosed with perinatal asphyxia had a 2.83-fold higher hazard of dying compared to those who were not asphyxiated (AHR = 2.83, 95% CI (1.75–4.58)). Similarly, neonates who suffered from respiratory distress syndrome had a 3 times higher hazard of death than those who did not (AHR = 3.01, 95% CI (1.80–5.01)). First minute APGAR score and birth weight were also independently associated with mortality on preterm neonates. Accordingly, this study revealed that each additional point of the APGAR score at the first minute of delivery was related to a 22% decrement of risk to neonatal mortality (AHR = 0.78, 95% CI (0.62–0.98)). Likewise, every 100 g birth weight addition was associated with an approximately 6.8% decrease in the hazard to death of preterm neonates (AHR = 0.32, 95% CI (0.17–0.58)).

## Discussion

In this cohort, the proportion of death among preterm neonates was 14.6% (95% CI (12.6–16.9%)) which is in line with the study conducted at Debretabor town health institutions, northwest, Ethiopia, 12.8%^24^. In contrast, this is lower than the finding from the study among preterm infants in different hospitals in Ethiopia where the proportion of preterm death ranged from 22.7 to 28.8%^12–14^. According to a study conducted by Muhe, the overall preterm death proportion was 22.7%. However, after excluding those who were not admitted to the NICU, the proportion of death in the unit rose to 28.8%. Therefore, the difference in the magnitude of death across studies in Ethiopia might be linked with selection bias, quality of care and study areas^12^. On the other hand, the proportion in our study is far from two findings in China where the overall mortality among preterm neonates was 1.9% and 5.0%^5,32^. The difference between the studies might be because of the study setting and socioeconomic status of the nations. Some of the studies are conducted in all pediatric departments where the risk of neonatal death may not be similar to those admitted only in the neonatal intensive care unit.

Approximately 1/3 and 4/5 of the deaths were recorded in the first day and the first week of life, respectively. This could be due to that many of the neonates was discharged from the hospital in the first two weeks of life even though the follow-up period ranged from 1 to 60 days. So, it is likely to see a greater number of deaths in the first week of life. This is consistent with a previous study conducted in Iran where, 39% and 84.3% of deaths were recorded in the first 24 h and the first week of the neonatal period, respectively^25^.

Preterm deliveries are often associated with underlying feto-maternal medical problems in the absence of spontaneous labor. Because of this, deliveries are conducted either by labor induction or CS^8^. Similarly, we found nearly half (52%) of the preterm neonates were born by CS. This is consistent with the study conducted by O. Gluck et al. where the proportion of preterm cesarean delivery was 53.1%^33^. In contrast, a recent multi-country survey found that the prevalence of women with preterm CS delivery was 36.7%^34^.

In this study, the factors associated with preterm mortality were fetal presentation, respiratory distress syndrome, perinatal asphyxia, birth weight, gestational age and APGAR score at the first minute. The risk of preterm neonatal mortality was approximately 2.3 times higher among neonates with a compound presentation. This finding was supported by a study conducted in Hawassa University Hospital, Ethiopia, where malpresentation was associated with death^35^. Malpresentation, together with other maternal complications and scarce resources could cause neonatal death, which is commonly observed in developing nations. Moreover, a fetus with compound presentation usually faces respiratory and circulatory anatomy collapses that could lead to death. Our finding also indicated that gestational age and neonatal mortality have an inverse relationship to each other. Accordingly, as the gestational age increased in 1 week, the risk of death among preterm neonates was decreased by 28%.

Every 100 g birth weight addition was associated with an approximately 6.8% decrease in the hazard to death of preterm neonates. This finding has been supported by various studies employed in Ethiopia, Egypt, Trinidad and Tobago, and China^5,6,15,35,36^. Therefore, it seems rationale that neonates with relatively low birth weight and various comorbid diseases are probably at high risk of dying.

In addition, our study figured out that respiratory distress syndrome was an independent predictor of preterm neonatal mortality. Death can occur in association with poor pulmonary outcomes and other obstetric reasons. Also, poor clinical setup, limited respiratory support devices and other resource scarcity issues in the hospital might contribute to raising the number of deaths associated with respiratory distress syndrome. In connection with this, neonates who were diagnosed with perinatal asphyxia had 2.83 hazards of dying compared to those who were not asphyxiated which has been also reported in China^5^.

### Limitation of the study

The study was not without limitations due to its retrospective nature and incomplete medical records. Primarily, it has limited maternal data that might add value to the existing findings. Secondly, there was possible selection bias as early deaths often lead to incomplete data and subsequent exclusion from the study. Thirdly, data on the site of delivery, home versus in the hospital, were incomplete. Lastly, the possibility of medical record errors and diagnostic subjectivity was not controlled.

## Conclusions

The survival probability of preterm neonates showed a significant decrement in the first week of life. Hypothermia, neonatal sepsis, respiratory distress syndrome, hypoglycemia, jaundice, perinatal asphyxia, seizure and meningitis were recorded as the frequent morbidities in preterm neonates. The proportion of neonatal death was lower than that of the other studies in Ethiopia. Furthermore, the most frequent morbidities associated with preterm neonatal death were respiratory distress syndrome and neonatal sepsis. Fetal presentation, gestational age, birth weight, first minute APGAR score, perinatal asphyxia and respiratory distress syndrome have been found as independent predictors of mortality in preterm neonates.

## Data Availability

The datasets used and/or analysed during the current study are available from the corresponding author on reasonable request.
